# Bacterial species cultured after electrofulguration in women with a history of antibiotic-recalcitrant urinary tract infections frequently compare with pre-fulguration findings: a pilot study

**DOI:** 10.1128/spectrum.04311-23

**Published:** 2024-07-09

**Authors:** Samuel B. Kusin, Ethan M. Fan, Alana L. Christie, Philippe E. Zimmern

**Affiliations:** 1 Department of Urology, University of Texas Southwestern Medical Center, Dallas, Texas, USA; 2 Simmons Comprehensive Cancer Center Biostatistics, University of Texas Southwestern Medical Center, Dallas, Texas, USA; Houston Methodist Hospital, Houston, Texas, USA

**Keywords:** electrofulguration, urinary tract infections, antibiotic resistance, urine cultures, women

## Abstract

Electrofulguration (EF) of areas of chronic cystitis in women with antibiotic-recalcitrant recurrent urinary tract infections (RUTIs) can result in improvement of their urinary tract infections (UTIs). We compared urine culture (UC) findings in patients before and after EF, as well as how they vary with cystitis stage at the time of EF, to evaluate for persistent species. After obtaining institutional review board approval, we retrospectively reviewed a prospectively maintained database of EF patients for those with positive UC findings in the 3–6 months preceding EF. Patient pre-EF UC was then compared with first positive UC after EF prompted by a new symptomatic UTI episode, with the hypothesis that the same species will be identified before and after EF. Exclusion criteria included UC from outside institution, neurogenic bladder, and need for catheterization. Ninety-nine women with pre- or post-EF UC-recorded organisms met the study criteria. The median age was 65 years (interquartile range 64–74), with a median time to first positive culture following fulguration of 9.7 months. For 26 patients with positive cultures both pre- and post-EF, the same organism was present in both cultures in 73% of the patients, with predominantly *Escherichia coli*. EF was effective at reducing the rate of UTIs in this population. For women undergoing EF for antibiotic-recalcitrant RUTIs and associated chronic cystitis lesions, 73% of those with a UC obtained at the time of a first symptomatic recurrent UTI episode post-EF expressed the same organism as before EF. Further study is needed to better understand the evolution of the microbiome post-EF.

**IMPORTANCE:**

Among women who experience a recurrent urinary tract infection after a fulguration procedure on areas of chronic cystitis in their bladder, there are no data available on whether the bacterial species found in urine cultures are the same or different from those present before fulguration. By removing the inflamed surface layer of cystitis during fulguration, it is possible that the procedure unmasks deep-seated bacteria. The bacterial kingdom in the bladder wall of these chronically infected women may be different from what is expressed sporadically in urine cultures. Confirming prior studies, we found that fulguration in women with antibiotic-recalcitrant recurrent urinary tract infections and cystitis lesions was effective at reducing the rate of urinary tract infections. At the time of a first symptomatic recurrent UTI episode post-fulguration, 73% expressed the same organism in urine culture as before fulguration. Further study is needed to better understand the evolution of the microbiome post-EF. This article evaluates persistent infections after electrofulguration of areas with chronic cystitis in post-menopausal women with antibiotic-recalcitrant recurrent urinary tract infections. Pre-fulguration urine cultures were compared with the first positive urine culture prompted by a new symptomatic UTI episode after electrofulguration, with the hypothesis that the same species will be identified before and after the fulguration procedure. Electrofulguration was effective at reducing the rate of UTIs in this population. However, 73% of those with a urine culture obtained at the time of a first symptomatic recurrent UTI episode post-electrofulguration expressed the same organism (predominantly *Escherichia coli*) as before the fulguration procedure. Further study is needed to better understand the evolution of the microbiome after electrofulguration.

## INTRODUCTION

Urinary tract infections (UTIs) are the most common outpatient infections in women, with over half experiencing at least one UTI in their lifetime (1, 2). Up to 40% of women who have an initial UTI episode are likely to have another episode, with 25%–40% of these women expected to experience recurrent UTIs (3–5). Recalcitrant recurrent urinary tract infections (RUTIs) present a treatment challenge due to frequent antibiotic use among these women, resulting in the development of antibiotic-recalcitrant infections (6–8). In addition, RUTIs have a significant economic and personal impact on women, resulting in a major decrease in their quality of life (9). The management of this condition varies with current guidelines recommending the use of daily low-dose suppression antibiotics, post-coital antibiotics, or self-starter antibiotics as first line therapies (10). Given the high prevalence of RUTIs and an increasing antimicrobial allergy and resistance trend, there is a need for effective non-antibiotic options in the management of RUTI (6, 7, 11).

We have previously reported on the efficacy of an electrofulguration (EF) procedure to eliminate chronic sites of infection inside the bladder in antibiotic-recalcitrant patients who would otherwise be candidates for intravenous (IV) antibiotic therapy or cystectomy (12–14). The use of EF is based on research demonstrating the presence of resident bacteria inside the bladder wall of post-menopausal women with chronic cystitis sites and RUTIs (15–18). In one study, women with antibiotic-refractory RUTI who underwent EF were found to have a 50%–70% improvement and cure rate of their RUTI compared to women who received antibiotic treatment alone (12, 19). This favorable evolution of their RUTI pattern has been linked to the eradication of chronic sites of cystitis in the bladder wall, with the success of this therapy enhanced when these inflamed areas are confined to the trigone, yet still observed but at a reduced rate when the whole bladder is involved as in the case of pancystitis (14, 20).

For women who have recurrent UTIs following EF, there are currently no data available on whether the urinary microorganisms found on urine cultures (UCs) in these women are the same or different from those present before EF. By removing the surface layer during EF, it is possible that the procedure unmasks deep-seated bacteria (15). The bacterial kingdom in these chronically infected women may be different from what is expressed sporadically in urine cultures in which polymorphism is frequently observed (15). In this study, we report on urine culture findings in patients before and after EF and on how they vary with the stage of cystitis noted at the time of the EF procedure.

## MATERIALS AND METHODS

A prospectively maintained database of women treated with EF for RUTIs at our institution was retrospectively reviewed. All women were treated by a single female pelvic medicine and reconstructive surgery (FPMRS) urologist practicing at a tertiary care urology facility. Inclusion criteria for this study included women with any positive UC finding preceding EF and preferentially those with positive UC in the 3–6 months prior to EF. Exclusion criteria included patients with a UC from an outside institution, neurogenic bladder, and/or need for catheterization. In addition, urine cultures were excluded with low count or contamination.

All women eligible for EF had a history of RUTIs defined as symptomatic UTI episodes confirmed by UC, with ≥2 UC in 6 months or ≥3 UC in a year (21). Prior to EF, women failed multiple UTI-directed treatment options, including culture-specific oral or IV antibiotic courses, as well as other preventative measures such as oral supplements, topical hormone replacement therapy, coital prophylaxis, and/or suppressive antibiotics (10, 22).

Prior to undergoing EF, women underwent a thorough urologic evaluation to detect unsuspected upper and/or lower urinary tract etiologies for their RUTIs. Pelvic examination looking for stage 2 or more pelvic organ prolapse and post-void residuals by bladder scan of >100 mL were performed. Imaging included renal scan or CT scan for any upper tract anomaly, and voiding cystourethrogram to evaluate for possible bladder/urethral pathologies (23).

As part of their evaluation, an in-office flexible cystoscopy was also performed under local anesthesia as previously described by Ordonez et al. (24) During this study, the urethra, bladder neck, bladder base, and the remainder of the bladder, with retroflexion evaluation of the trigone, were inspected for areas of chronic cystitis, and all lesions were documented with photographs (24). The extent of bladder involvement was categorized by stage, with stage 1 involving the trigone alone; stage 2 involving the trigone and bladder base; stage 3 involving the trigone, bladder base, and the bladder walls lateral to the ureteric orifices; and stage 4 involving the entire bladder (pan-cystitis) (24, 25). Patients were considered to have antibiotic-recalcitrant RUTIs once they developed antibiotic allergies and had at least three or more classes of antibiotic resistance since this combination of allergy and resistance further complicated their antibiotic care due to limited options, sometimes relying only on IV antibiotic drugs.

The choice of an EF procedure was considered in an RUTI patient with a normal upper and lower urinary tract evaluation but endoscopic evidence of chronic cystitis lesions amenable to fulguration, with the assumption that these chronic lesions harbored bacteria provoking these recurrences (15). EF procedures were carried out in an outpatient setting by a single surgeon as previously described (12, 19). In brief, cystoscopy was performed under anesthesia with a 17.5-Fr female urethrocystoscope. Using a fine-tip monopolar bugbee electrode on a low setting (20–25), all inflamed lesions were superficially fulgurated. Patients were discharged home on the same day. Post-operative irritative bladder symptoms were treated with fluid limitation and topical analgesics. Most patients were treated with daily low-dose antibiotic suppression for 6 weeks following EF to avoid a new UTI during the first stages of healing. At their 6 weeks of follow-up visit, based on their UTI symptomatology and urinalysis findings, this preventive antibiotic course is generally discontinued. Patients are prescribed antibiotics to have available for self-therapy in case UTI symptoms recur in the coming months. They are also provided with a standing order for a urine culture to be obtained prior to re-starting on any antibiotic treatment. The first symptomatic UTI following EF, with a positive UC, was recorded. Approximately 6 months following EF, an office flexible cystoscopy was repeated to confirm the completion of the healing process over the fulgurated sites and to look for new areas of bladder wall inflammation.

The primary outcome was urine culture results for the first symptomatic UTI after EF. All UC results were obtained from our university microbiology laboratory. We classified patients into three groups according to UC results available pre- and post-EF: group 1 had both pre- and post-EF cultures; group 2 had pre-EF cultures only; and group 3 had post-EF cultures only. Demographic information, medical history, primary urinary symptoms, UC, and cystoscopy findings were obtained and reviewed from the electronic medical record (EMR) (EPIC) by an independent reviewer not involved in the care of these patients. Sexual activity was self-reported.

### Statistical methods

Descriptive statistics were provided as medians and interquartile ranges (IQRs) for continuous measures and as frequencies and percentages for categorical measures. We tested for associations between the availability of positive cultures and categorical patient characteristics using Fisher’s exact test. Analysis of variance was used to test for differences in continuous patient characteristics by availability of positive cultures. All tests were performed at the 0.05 significance level without adjustment for multiple comparisons using SAS version 9.4 (SAS Institute Inc., Cary NC).

## RESULTS

From 2004 to 2020, 99 of 108 patients met all study criteria. Nine patients were excluded due to urine culture showing contamination or low bacterial counts. The median age was 65 years (IQR 64–74). Table 1 indicates patient clinical characteristics by cultures available. Baseline characteristics were similar between groups with the majority of women being menopausal, Caucasian, and having stage 1 cystitis (68%). Thirteen patients (13%) were diabetics and 28 (28%) were on menopausal replacement therapy. The most common primary lower urinary tract symptom reported with a urinary tract infection was urinary frequency. The median time to first positive culture following fulguration was 9.7 months (IQR 4.8–19.8). Thirty-four percent of patients reported that they were sexually active (34 of 99 who reported on sexual activity). We did not see any association between the stage of cystitis at initial cystoscopy and having a positive UC after EF (*P* = 0.7).

**TABLE 1:** Patient characteristics by urine cultures available, pre-, post- or pre-/post-EF

	Pre- and post-EF (*n* = 26)	Pre-EF only (*n* = 47)	Post-EF only (*n* = 26)	P
Cystitis stage				
1	20 (76.9)	29 (61.7)	18 (69.2)	0.9
2	1 (3.8)	5 (10.6)	3 (11.5)	
3	4 (15.4)	9 (19.1)	3 (11.5)	
4	1 (3.8)	4 (8.5)	2 (7.7)	
Median age (years) (IQR)	70 (58–73)	68 (57–74)	61 (47–77)	0.3
Median body mass index (IQR)	24 (21–31)	24 (21–30)	24 (23–27)	0.8
Race				
White	23 (88.5)	42 (89.4)	21 (80.8)	0.5
Black/African American	0 (0.0)	0 (0.0)	2 (7.7)	
Hispanic	1 (3.8)	3 (6.4)	1 (3.8)	
Asian	1 (3.8)	2 (4.3)	2 (7.7)	
Other	1 (3.8)	0 (0.0)	0 (0.0)	
Median gravidity (IQR)	3 (2–3)	2 (2–3)	2 (0–3)	0.6
Median parity (IQR)	2 (2–3)	2 (2–3)	1 (0–3)	0.13
Diabetes				
None	22 (84.6)	39 (83.0)	25 (96.2)	0.5
Adult onset	2 (7.7)	5 (10.6)	0 (0.0)	
Insulin dependent	2 (7.7)	3 (6.4)	1 (3.8)	
Median Aa point, −3 to 3 (IQR)	−3 (−3 to −2)	−3 (−3 to −2)	−3 (−3 to −2)	0.9
Median Ap point, −3 to 3 (IQR)	−3 (−3 to −2)	−3 (−3 to −3)	−3 (−3 to −2)	0.5
Primary symptom				
Frequency	16 (61.5)	34 (72.3)	22 (84.6)	0.069
Urgency	0 (0.0)	2 (4.3)	1 (3.8)	
Nocturia	1 (3.8)	0 (0.0)	0 (0.0)	
Dysuria	6 (23.1)	3 (6.4)	1 (3.8)	
Incontinence	0 (0.0)	5 (10.6)	2 (7.7)	
Other	3 (11.5)	3 (6.4)	0 (0.0)	
Urgency	16 (64.0)	42 (91.3)	19 (90.5)	0.012
Straining	9 (37.5)	18 (40.9)	10 (47.6)	0.8
Nocturia	16 (64.0)	36 (78.3)	22 (91.7)	0.065
Incomplete emptying	12 (52.2)	29 (61.7)	11 (57.9)	0.7
Incontinence type				
None	2 (13.3)	3 (15.0)	2 (18.2)	0.9
Stress	9 (60.0)	9 (45.0)	7 (63.6)	
Urge	3 (20.0)	5 (25.0)	2 (18.2)	
Mixed	1 (6.7)	3 (15.0)	0 (0.0)	
Hysterectomy	11 (52.4)	21 (50.0)	13 (61.9)	0.7
Oophorectomy	6 (30.0)	16 (36.4)	12 (52.2)	0.3
Menopausal	16 (66.7)	37 (80.4)	13 (54.2)	0.070
Hormone replacement therapy				
None	18 (69.2)	33 (70.2)	20 (76.9)	0.9
Systemic	1 (3.8)	3 (6.4)	1 (3.8)	
Local	7 (26.9)	11 (23.4)	5 (19.2)	
Sexually active	11 (52.4)	15 (33.3)	8 (42.1)	0.3
History of tobacco use	4 (19.0)	6 (13.3)	4 (23.5)	0.6

For 26 patients with positive cultures both pre- and post-EF (Table 2), the same organism was present in both cultures in 73% (19 of 26) of these patients. *Escherichia coli* was found in 12 patients, *Enterococcus faecalis* in 4 patients, Gram-negative rods in 2 patients, and *Klebsiella pneumoniae* in 1 patient. The median time between pre- and post-EF cultures was 1.2 years (IQR 0.7–2.2). Of note, before fulguration, 4 UCs had polymorphism compared to 12 UCs after EF. Eight of the 12 patients with *E. coli* infections pre- and post-EF had resistance profiles available, including 4 who had the same resistance profile, 2 who developed new resistances post-EF, and 2 who had fewer resistances post-EF. No significant association was found between stage of cystitis and presence of the same bacteria pre- and post-EF, though all five patients with cystitis at stages 3 and 4 did have the same bacterial strain.

**TABLE 2:** Organisms present pre- and post-EF (*n* = 26)^*a*^

Pre-EF	Post-EF	*n* (%)
*E. coli*	*E. coli*	4
*E. coli*	*E. coli + E. faecalis*	3
*E. coli*	*E. coli + Proteus*	1
*E. coli*	*E. coli* + GNR + *K. pneumoniae*	1
*E. coli*	*E. faecalis + Staph*	1
*E. coli + E. faecalis + K. pneumoniae*	*E. coli*	1
*E. coli + Enterobacter + K. pneumoniae*	*E. coli*	1
*E. coli + E. faecalis*	*E. coli*	1
*E. faecalis*	*E. faecalis + E. coli*	1
*E. faecalis*	*E. faecalis + E. coli* + GNR	1
*E. faecalis*	*E. faecalis + E. coli* + GNR + *K. pneumoniae + Citro*	1
*E. faecalis*	*E. coli*	1
*E. faecalis*	*K. pneumoniae*	1
*E. faecalis*	*E. coli + K. pneumoniae*	1
*E. faecalis*	*E. coli* + GNR + *Morganella*	1
*E. faecalis + E. coli*	*E. faecalis* + GNR + *Proteus*	1
GNR	GNR	1
GNR	GNR + *Proteus*	1
GNR	*E. coli*	2
*K. pneumoniae*	*K*. *pneumoniae + E. coli*	1

^*a*^ *Citro*, *Citrobacter*; GNR, Gram-negative rod; *Staph*, *Staphylococcus*; Proteus: *Proteus mirabilis*.

In Table 3, the frequencies of each organism for patients with only pre- or post-EF UC available are compared to patients with both pre- and post-EF UC available. The comparison was similar for each group with no significant differences found. The frequency of *Enterococcus faecalis* present was most different, with 19% of pre-EF only UC positive for *Enterococcus faecalis* compared to 38% of cultures for patients with both pre- and post-EF UC available (*P* = 0.096). For patients with only post-EF cultures available, 8% of the patients had cultures positive for *Enterococcus faecalis* compared to 31% of patients who had pre- and post-EF cultures available (*P* = 0.075).

**TABLE 3:** Comparison of organisms by culture availability^*a*^

	Pre-EF culture			Post-EF culture		
	Pre-only, *n* (%)	Pre and post, *n* (%)	*P*	Post only, *n* (%)	Pre and post, *n* (%)	*P*
*E. coli*	25 (53)	14 (54)	>0.9	18 (69)	21 (81)	0.5
*Klebsiella*	11 (23)	3 (12)	0.4	7 (27)	5 (19)	0.7
*Enterococcus*	9 (19)	10 (38)	0.096	2 (8)	8 (31)	0.075
GNR	2 (4)	4 (15)	0.18	3 (12)	7 (27)	0.3
*Proteus*	–^*b*^	–		2 (8)	3 (12)	>0.9
*Citrobacter*	–	–		1 (4)	1 (4)	>0.9
*Enterobacter*	0 (0)	1 (4)	–	–	–	
*Morganella*	–	–		0 (0)	1 (4)	–
*Pseudomonas*	1 (2)	0 (0)	–	1 (4)	0 (0)	–
*Serratia*	–	–		1 (4)	0 (0)	–
*Staphylococcus*	7 (15)	0 (0)	0.046	5 (19)	1 (4)	0.19

^*a*^ The pre-EF culture section compares the pre-EF culture results from patients with both pre- and post-EF cultures to patients with only the pre-EF culture. Similarly, the post-EF culture section compares the post-EF culture results from patients with both pre- and post-EF cultures to patients with only post-EF culture.

^*b*^ No proteus strain present.

## DISCUSSION

The American Urological Association guidelines for management of RUTI recommends treatment of each UTI based on urine culture sensitivities. The guidelines then suggest a progressive approach that may include daily low-dose antibiotic therapy, supplements such as cranberry, D-mannose, methenamine hippurate, and vaginal estrogen for peri- and post-menopausal women (10). In regard to vaginal estrogen, although most patients who were using hormonal replacement therapy (HRT) were on local therapy, 72% of the women were not on any type of therapy. The low-level use of vaginal hormones in our population of post-menopausal women is attributed to several factors, including cost, minimal motivation due to lack of sexual activity, expressed concerns about risk of breast cancer, and prior lack of efficacy to prevent recurrent UTI episodes. The need for alternatives to antibiotics to treat RUTI is well established. The cohort represented in this study is challenging to treat, given the recalcitrant nature of these infections to antibiotic treatment as well as their high levels of antibiotic resistance and antibiotic allergies (7).

This study is the first to provide detailed information on the bacterial composition of urine in women who underwent EF for antibiotic-recalcitrant RUTIs with various stages of cystitis. Our understanding of the use of EF in the management of RUTI is rapidly evolving. The existing literature on EF is largely limited to reporting outcomes of the procedure based on the recurrence of UTI. As longer-term data become available, it is clear that EF is not effective for everyone (26). The reasons for both the success and failure of EF are still being investigated. Understanding the reasons for success and failure is critical to better characterize the population which might benefit from this procedure. While the results of this study do support previous findings that many patients become UTI-free following EF, our novel finding that 73% of patients who failed treatment and had positive cultures both before and after EF had the same species in both cultures leads to questions about why these bacteria are recurrent.

The underlying mechanism of RUTI is currently the subject of different hypotheses. One hypothesis, the intracellular bacterial community (IBC)-quiescent intracellular reservoir (QIR) theory, postulates that uropathogenic bacteria form IBCs within the bladder epithelium, making them recalcitrant to antibiotic treatment (15, 16). These IBCs rapidly multiply and invade other cells. Uropathogenic bacteria may also establish QIRs of non-replicating bacteria in the bladder epithelium (27, 28). An alternative hypotheses for RUTI states that reinfection may occur from the translocation of resident gut uropathogens into the urinary tract (28).

Previously, our group has shown that it is not always possible to identify areas of infection during cystoscopy (29). In a study where cold-cut bladder biopsies were collected from both areas of active cystitis and areas with no cystitis, 50% of no cystitis biopsies had immunofluorescent staining patterns suspicious for infection (29). One possible reason for RUTI after EF is that QIRs that are not visible on cystoscopy may activate at a later time, leading to reinfection. Additionally, in patients with high bladder stages (stages 3 and 4), it is often not possible to fulgurate all visible areas of chronic cystitis, resulting in the need for repeat fulguration (14) when the UTI episodes continue after the first EF.

Six months following EF, women underwent a repeat in office cystoscopy to verify healing of fulguration sites and to check for any new areas of cystitis that might have surfaced over the bladder wall. At these new sites or, in the case of pan-cystitis, at sites that were not able to be fulgurated the first time around, bacteria may continue to be produced. These bacteria, as indicated by our results, appear to be similar to the species present on UC prior to EF, though this observation would need to be confirmed with more elaborate bacterial sequencing. In addition, during EF, it is possible that bacteria deeper in the bladder wall become unmasked, providing a possible explanation for the increased polymorphism observed on UC following EF. This is supported by findings by De Nisco et al., who used florescent *in situ* hybridization for bladder biopsies of women with RUTIs at the time of their original EF and reported that more bacterial species were present on tissue cultures than on UC (15). For women who have positive cultures post-EF and remain symptomatic, repeat EF has shown an additional cure/improvement benefit (13).

Strengths of this study included evaluation and treatment by a single FPMRS surgeon at a tertiary referral practice. Patient evaluation and treatment plans followed were consistent between patients. Study limitations included difficulty in obtaining a larger number of patients who met the study criteria. This was likely due to the success of EF for the post-EF UC, as well as limited pre-EF UC data because some women on prolonged low-dose daily antibiotic suppression before EF had no pre-EF UC data available. Since we excluded urine cultures that were performed at outside facilities and were not recorded in the EMR, we also limited our pre-EF UC information. Furthermore, it was noted that our UC data were mainly for women with early-stage cystitis; thus, our findings may not be generalizable to all women with antibiotic-recalcitrant RUTI (14). Additionally, the lack of phenotypic and genomic information for both pre- and post-EF bacterial species limits our findings. Finally, data on peri-coital prophylaxis usage were limited due to the low level of sexual activity in this patient population and could not be adequately reported.

### Conclusion

In women undergoing EF for antibiotic-recalcitrant RUTIs and associated chronic cystitis lesions, 73% of those with a UC at the time of a first symptomatic UTI episode post-EF expressed the same pre-EF species. Further study is needed to better understand the nature and evolution of the microbiome post-EF.

## Data Availability

The data sets generated and/or analyzed during the current study are available from the corresponding author on reasonable request.
